# Vitamin D Binding Protein and the Biological Activity of Vitamin D

**DOI:** 10.3389/fendo.2019.00718

**Published:** 2019-10-24

**Authors:** Rene F. Chun, Albert Shieh, Carter Gottlieb, Vahe Yacoubian, Jeffrey Wang, Martin Hewison, John S. Adams

**Affiliations:** ^1^Department of Orthopaedic Surgery, David Geffen School of Medicine at UCLA, Los Angeles, CA, United States; ^2^Department of Medicine, David Geffen School of Medicine at UCLA, Los Angeles, CA, United States; ^3^Institute of Metabolism and Systems Research, University of Birmingham, Birmingham, United Kingdom

**Keywords:** vitamin D, free vitamin D, bone, immunology, DBP, CYP27B1, VDR

## Abstract

Vitamin D has a long-established role in bone health. In the last two decades, there has been a dramatic resurgence in research interest in vitamin D due to studies that have shown its possible benefits for non-skeletal health. Underpinning the renewed interest in vitamin D was the identification of the vital role of intracrine or localized, tissue-specific, conversion of inactive pro-hormone 25-hydroxyvitamin D [25(OH)D] to active 1,25-dihydroxyvitamin D [1,25(OH)_2_D]. This intracrine mechanism is the likely driving force behind vitamin D action resulting in positive effects on human health. To fully capture the effect of this localized, tissue-specific conversion to 1,25(OH)_2_D, adequate 25(OH)D would be required. As such, low serum concentrations of 25(OH)D would compromise intracrine generation of 1,25(OH)_2_D within target tissues. Consistent with this is the observation that all adverse human health consequences of vitamin D deficiency are associated with a low serum 25(OH)D level and not with low 1,25(OH)_2_D concentrations. Thus, clinical investigators have sought to define what concentration of serum 25(OH)D constitutes adequate vitamin D status. However, since 25(OH)D is transported in serum bound primarily to vitamin D binding protein (DBP) and secondarily to albumin, is the total 25(OH)D (bound plus free) or the unbound free 25(OH)D the crucial determinant of the non-classical actions of vitamin D? While DBP-bound-25(OH)D is important for renal handling of 25(OH)D and endocrine synthesis of 1,25(OH)_2_D, how does DBP impact extra-renal synthesis of 1,25(OH)_2_D and subsequent 1,25(OH)_2_D actions? Are their pathophysiological contexts where total 25(OH)D and free 25(OH)D would diverge in value as a marker of vitamin D status? This review aims to introduce and discuss the concept of free 25(OH)D, the molecular biology and biochemistry of vitamin D and DBP that provides the context for free 25(OH)D, and surveys *in vitro*, animal, and human studies taking free 25(OH)D into consideration.

## Introduction

The benefits of vitamin D for mineral homeostasis and bone health are well-established. Deficiency of vitamin D, rickets in children and osteomalacia in adults, can be treated or prevented with oral supplements of vitamin D. Despite promising pre-clinical observations and vitamin D-deficiency association studies, the impact of vitamin D on other aspects of human health such as common cancers, cardiovascular disease, type 2 diabetes obesity, autoimmune disorders, and infectious disease remains controversial (1–5). Randomized, controlled supplementation trials are required to better define the extra-skeletal roles of vitamin D. However, these trials are complicated by two unanswered questions: (1) what is the best marker of vitamin D status and (2) what constitutes a level that is sufficient to promote the health benefits of vitamin D?

## Endocrine Vitamin D Metabolism and Action

**Figure 1:**
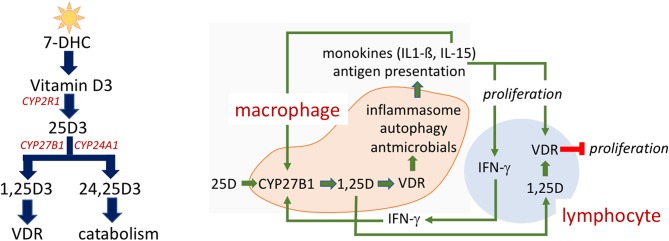
Vitamin D metabolism and action. Vitamin D metabolites **(Left)**. 7-dehydrocholesterol (7-DHC) is photoconverted to vitamin D_3_ by UV exposure of skin. CYP2R1 in the liver hydroxylates vitamin D_3_ to 25-hydroxyvitamin D_3_ (25(OH)D). Another hydroxylation to 25(OH)D_3_ by CYP27B1 occurs in the kidney but also in a number of extra-renal tissues produces the active 1,25-dihydroxyvitamin D_3_ (1,25(OH)_2_D_3_). 1,25(OH)_2_D_3_ is the cognate ligand for the vitamin D receptor (VDR), a nuclear transcription factor, that directs 1,25(OH)_2_D_3_ regulated gene transcription. CYP24A1 is responsible for the hydroxylation that yields 24,25-dihydroxyvitamin D (24,25(OH)_2_D_3_) and subsequent catabolism to non-biologically active metabolites. Intracrine mechanism in immune cells **(Right)**. Vitamin D action in immune cells is reliant upon the intracrine (local) production of active 1,25(OH)2D within the macrophage. Vitamin D status of the host as defined by serum, extracellular 25(OH)D levels impact immune response, as 25(OH)D is the substrate for CYP27B1. A complex interplay between monokine signaling, that can both be responsive to and stimulatory of 1,25(OH)_2_D synthesis, and regulatory signaling among innate and adaptive immune cells is shown.

The name “vitamin D” in this review refers to a collection of secosterol molecules detectable in the serum of vertebrates (left panel, Figure 1). Briefly, cholecalciferol or vitamin D3 (vitamin D) results from the ultraviolet B (UVB; 290–315 nm)-mediated photolytic-conversion of 7-dehydrocholesterol (DHC) in skin (6–8). Vitamin D can also be obtained from (i) food, principally from fortified dairy and juice products, (ii) consumption of fresh caught fish (e.g., salmon) (9), and (iii) oral vitamin D supplements. Vitamin D2 (ergocalciferol) is naturally found in fungi (e.g., mushrooms) and sometimes used in food fortification and supplementation regimes. Regardless vitamin D2 proceeds through the same modifications as described for vitamin D3 below.

Once in the general circulation vitamin D is bound to its serum carrier, vitamin D binding protein (DBP) and to a lesser extent albumin (10, 11), and is subject to a first hydroxylation step by vitamin D substrate-dependent 25-hydroxylase [CYP2R1 (12) and possibly a yet unidentified hydroxylase(s) (13)] in the liver resulting in 25-hydroxyvitamin D (25(OH)D). Very little 25(OH)D (~5%) is secreted in to the bile (14). Rather, the bulk of 25(OH)D re-enters the circulation, once again bound to either DBP or albumin for endocrine transport to target tissues. DBP- and albumin-bound 25(OH)D in urine is reclaimed by tubular epithelial cells in the kidney (15). Here internalized 25(OH)D is freed from its carrier protein(s), becoming substrate for (i) the low capacity 25-hydroxyvitamin D-1-alpha-hydroxylase (CYP27B1) and production of the active, hormonal form of vitamin D, 1,25-dihydroxyvitamin D (1,25(OH)_2_D) or (ii) the high calpacity 25-dihydroxyvitamin D-24 hyxdoxylase (CYP24A1) to form the largely non-biologically active metabolites 24,25-dihydroxyvitamin D (24,25(OH)_2_D) and 1,24,25-trihydroxyvitamin D, respectively (16). The various hydoxylated forms gain access to the general circulation bound to DBP or albumin. Rheostatic endocrine control over the reciprocal production of 1- and 24-hydroxylated vitamin D metabolites is exerted by parathyroid hormone and FGF23. Parathyroid hormone increases activity of CYP27B1-hydroxylase (17, 18), decreases product output by CYP24A1 (19, 20); thus, increases the “activation” quotient of product 1,25(OH)_2_D:substrate 25(OH)D in the serum. On the other hand, FGF-23 blunts CYP27B1 activity (21) and promotes CYP24A1 activity; thus, decreases the 1,25(OH)_2_D:25(OH)D activation quotient and increases the 24,25(OH)_2_D:25(OH)D inactivation quotient (22, 23).

As noted above, 1,25(OH)_2_D can be chaperoned in an endocrine mode to potential target tissues that employ serum bound, extracellular 1,25(OH)_2_D as a specific ligand for transactivation of the vitamin D receptor (VDR) in the target cell driving 1,25(OH)_2_D-VDR directed differential gene expression (24). A major caveat in the concept of direct endocrine action of 1,25(OH)_2_D is the fact all adverse physiological consequences of vitamin D deficiency in humans are associated with a low serum 25(OH)D, not a low 1,25(OH)_2_D level [Table 1; (25)]. In fact, in the basal state, before treatment to raise 25D levels, subjects with low serum 25D often have serum 1,25D levels that are relatively elevated as a consequence of compensatory secondary hyperparathyroidism. In this instance the increase in the host's circulating concentration of PTH drives an increase in renal 1,25D production. This suggests that 25D deficiency in the serum is a cause for “endocrine resistance” to circulating levels of the 1,25D hormone at the level of the gut. After vitamin D restoration treatment with return of 25D balance to normal and resolution of secondary hyperparathyroidism, there is an increase in intestinal calcium absorption even though there is a relative decrease, or no change in the circulating serum 1,25D level. This suggests that there may be local conversion of 25D to 1,25D in the gut outside of the serum compartment that is driving intestinal calcium absorption and/or that only measuring the total amount of vitamin D metabolite(s) in the serum, may be an inadequate biomarker of response to restoration of 25D levels in the blood to normal.

**Table 1:** Vitamin D and human health.

**Adverse health outcomes significantly associated with low serum 25(OH)D level**	
Low bone density	Obesity
Hip fractures	Insulin resistance
Non-vertebral fractures	Type 1 diabetes
Heart attack	Type 2 diabetes
Hypertension	Cancer
Stroke	Preterm delivery
Neurocognitive dysfunction	Pre-eclampsia
Proximal muscle weakness	Inflammation/infection
Autoimmune diseases	Multiple sclerosis

*Numerous health conditions have been associated with low total serum levels of 25(OH)D and are listed in the table. Conditions pertaining to bone health are indicated in blue. Table is based on reviews by Rosen et al. (2) and Rosen and Taylor (4)*.

## Local Vitamin D Metabolism and Action

In non-renal tissues, the CYP27B1 converts 25(OH)D to 1,25(OH)_2_D for local usage in paracrine, autocrine, and intracrine regulated activities (26). Perhaps the most relevant physiological/pathophysiological example of these events (e.g., those confined to the local tissue microenvironment outside of circulating serum compartment) are the human granuloma forming diseases like sarcoidosis and tuberculosis (27). In these disease states, cells of the innate immune response, principally macrophages, express the same metabolic machinery to synthesize 1,25(OH)_2_D intracellularly when presented with a CYP27B1 activating signal and with sufficient 25(OH)D in the extracellular space to serve as substrate for the CPY27B1. When the extracellular concentration of 25(OH)D falls below the equivalent of ~20 ng·mL^−1^ or 50 nM, the *intracrine* production of 1,25(OH)_2_D via the CYP27B1-hydroxylase becomes limiting; unlike the renal CYP27B1, the enzyme in the macrophage is highly substrate-drive (28). Taking the human granuloma-forming, macrophage dominant infectious disease tuberculosis (TB) as an example, in the face of deficient extracellular substrate 25(OH)D the macrophage CYP27B1 is unable to generate enough active 1,25(OH)_2_D metabolite to effectively ligand sufficient VDR in that cell to promote expression of vitamin D-dependent antimicrobial genes (29, 30). The end result is failure of the macrophage to mount an effective autophagy-related, vesicular killing response to ingested *Mycobacterium tuberculosis (M. tb)* (31, 32). *In vitro* this failure can be rescued in a 25(OH)D concentration-dependent fashion by exchanging vitamin D deficient human serum with vitamin D sufficient serum (>30 ng·mL-1 or 75 nM); in other words, rescue of the macrophage innate immune is achieved by conditioning activated macrophages *ex vivo* in serum from the same host after treatment of the host with vitamin D *in vivo* (30). Demonstrating successful rescue from 25(OH)D deficiency *in vivo* in humans exposed to or in the very early phases of infection with *M. tb*. would support the value of 25(OH)D-driven innate immune competence in prevention and use as adjunctive therapy early in the course of this disease (33).

The local antimicrobial capacity of the macrophage is subject to intracrine, autocrine, and paracrine feedforward and feedback immune regulatory circuits. This regulatory network is depicted schematically in the right panel of Figure 1. Stimulation of the Toll-like receptor signaling pathway by pathogen-associated molecular pattern (PAMP) molecules induces expression of CYP27B1 and VDR as well as of monokines (e.g., IL-15 and IL-1β). In an *autocrine* mode, these two monokines act to amplify expression of the CYP27B1 and 1,25(OH)_2_D-VDR-directed generation of antimicrobial peptides (34). In a *paracrine* fashion IL-1ß mobilizes and activates cells of the adaptive immune response (35–37). Activation of the Th1 subset of “helper” lymphocytes promotes: (1) production of IFN-γ, the most potent known stimulator of the macrophage CYP27B1-hydroxylase (38); and (2) induction of expression of the VDR in adaptive immune response cells (39, 40). When IFN-γ-driven production of 1,25(OH)_2_D in the macrophage is robust enough to allow escape of the active vitamin D metabolite into the local, pericellular inflammatory microenvironment, this 1,25(OH)_2_D is sufficient to drive VDR-dependent gene expression in activated lymphocytes such as inhibiting proliferation of those lymphocytes. As such, the predominant *paracrine* action of 1,25(OH)_2_D in this setting is to modify the adaptive immune response (41) and turn down IFN-γ and macrophage CYP27B1 gene expression, preventing a potential overzealous adaptive (auto)immune response harmful to the host. Therefore, 25(OH)D “sufficiency” in the serum of the host appears to be paramount in providing the optimal IFN-γ-mediated feedback control on 1,25(OH)_2_D synthesis by the macrophage and appropriate antimicrobial response to ingested microbes. For example, failure of this normal feedback control in disseminated infection with *M. tb*. may result in escape of 1,25(OH)_2_D from the local immune microenvironment into the serum tuberculosis, resulting in a form of endocrine-acting “1,25(OH)_2_D intoxication” and life-threatening hypercalcemia. This form of extra-renal 1,25(OH)_2_D intoxication can occur in certain granuloma-forming diseases such as tuberculosis (where pathogen is known) and sarcoidosis (where pathogen is unknown).

These scenarios indicate that the serum level of bioavailable 25(OH)D to macrophages is a key determinant of normal/abnormal physiological control of innate and adaptive immunity in the host with low serum 25(OH)D levels. Cell and molecular biology experiments have established co-existent expression of the CYP27B1 and VDR in the same cell (Table 2). This observation makes all of these cell-types potential candidates for intracrine metabolism and action of 1,25(OH)_2_D generated from 25(OH)D available to that cell from its extracellular microenvironment *in vivo*. Beyond tuberculosis just discussed, many human diseases are associated with “low” serum 25(OH)D levels compared to matched controls without the disease (Table 1). In cardiovascular disease, the leading cause of mortality in the US, mortality is inversely related to serum 25(OH)D <20 ng/ml (42, 43), though this is disputed (44). All of these disease states are associated with altered host immunity. Perhaps this is the common link to adverse outcomes in this otherwise diverse set of disorders.

**Table 2:** Cell types that express both CYP27B1 and VDR.

**Cells co-expressing a functional CYP27B1 and VDR**	
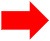 Macrophage	Enterocyte
Dendritic cell	Decidual stromal cell
Parathyroid cell	Fetal trophoblast
Osteoblast	Prostate epithelial cell
Osteoclast	Vascular endothelial cell
Keratinocyte	Pancreatic β cell
Mammary epithelial cell	Renal tubular cell

*In contrast to the endocrine action based on kidney production of 1,25(OH)_2_D that travels through the general circulation to VDR-possessing target cells, a number of cell types harbor both CYP27B1 and VDR making intracrine metabolism and action possible. The red arrow indicates the macrophage, the cell type in which the intracrine metabolism of 25(OH)D to 1,25(OH)_2_D and 1,25(OH)_2_D-directed gene expression has been most clearly demonstrated ex vivo*.

## Defining Vitamin D Status

The total serum 25(OH)D level (the sum of 25(OH)D that is bound to carrier proteins and is free in the circulation) is the currently accepted marker of choice for defining vitamin D status in any given individual. Its preferential use is based on (i) analysis of the existing body of clinical research, (ii) assay advances that confer easy and accurate measurements and (iii) the fact that it is the most abundant and stable of the various vitamin D metabolites in serum; 25(OH)D has a relatively long half-life (15 days) compared to 1,25(OH)_2_D (15 h). However, health organizations across the globe differ significantly in their definition of what level of total circulating 25(OH)D constitutes sufficiency and deficiency for a “normal” population without evidence of active bone disease (e.g., osteoporosis, hyperparathyroidism, etc.). In North America, the Institute of Medicine has recommended the value of ≥20 ng/ml 25(OH)D (50 nmol/L) for sufficiency (45), whereas the Endocrine Society recommended for ≥30 ng/ml (75 nmol/L) (46). The Vitamin D Council states that individuals should strive for levels above 40 ng/ml (100 nmol/L) (47). In contrast to this, the UK Science Advisory Council on Nutrition defined vitamin D deficiency as serum levels of 25(OH)D <10 ng/ml (25 nmol/L), but did not recommend an optimal level for human health (48). Although the 10 ng/ml level is held by some other European nations, some have recommended higher levels (49). Investigators in the vitamin D field have highlighted the limitations of the total 25(OH)D as the routine biomarker for vitamin D status in various commentary and review articles (50, 51) and discussed other potential markers and the possible need of a “vitamin D panel” (52, 53).

Human data indicates that the threshold for detection of a relative increase in the serum iPTH at the individual and population level occurs when the total serum 25(OH)D level falls below ~30 ng/ml (54, 55). Thus, PTH offers a measurable biological consequence of “low” 25(OH)D. However, PTH levels are not exclusively controled by 25(OH)D as serum concentration of ionized calcium is sensed at the parathyroid gland by the calcium-sensing receptor (CaSR). When serum calcium levels drop, CaSR signal transduction in the parathyroid yields an increase in PTH production that then enters the general circulation resulting in PTH's endocrine effects.

Another reason for the uncertainty concerning the validity of total 25(OH)D as an exclusive serum marker for vitamin D health is due to the complex molecular biology and biochemistry of 25(OH)D-associated bioactivities. This is particularly relevant to the intracrine, paracrine or endocrine conversion of 25(OH)D to active 1,25(OH)_2_D, because (i) the local concentrations vitamin D metabolites outside of the serum compartment cannot be easily measured *in vivo* and (ii) the subsequent molecular actions of 1,25(OH)_2_D in conjunction with its binding by the VDR is dependent on diverse mechanisms beyond simple variations in circulating 25(OH)D. These include: (1) the transport and target tissue uptake of 25(OH)D; (2) the directed intracellular transport of 25(OH)D to the inner mitochondrial to CYP27B1 for enzymatic conversion of 25(OH)D to 1,25(OH)_2_D; (3) export if 1,25(OH)_2_D from the mitochondia and binding of 1,25(OH)_2_D to VDR; and (4) competing catabolism of 1,25(OH)_2_D by the enzyme CYP24A1 also located on the inner mitochondrail membrane. In this review, we will briefly discuss the molecular biology and biochemistry behind these processes, with particular emphasis on the role of free 25(OH)D as a key determinant of the downstream actions of 1,25(OH)_2_D, specifically in bone and mineral health.

## Molecular Biology and Biochemistry of Vitamin D Action

1,25(OH)_2_D is the active vitamin D molecule with 25(OH)D being its immediate precursor (panel A, Figure 1). It is 1,25(OH)_2_D that drives vitamin D-regulated gene expression in target cells. Under normal conditions, the level of serum 1,25(OH)_2_D is tightly regulated within a narrow range (30–60 pg/ml) at a level that is 1,000X less plentiful than its 25(OH)D precursor. In non-pregnant humans, 1,25(OH)_2_D in the serum comes almost exclusively from expression of CYP27B1 in the kidney. Circulating 1,25(OH)_2_D's endocrine actions are to regulate the serum level of calcium by optimizing intestinal calcium absorption and/or calcium resorption from the skeleton (56). Not surprisingly then, when kidney failure occurs, there is (i) an accompanying decrease in renal CYP27B1 capacity, (ii) a fall in 1,25(OH)_2_D production and (iii) a reduction in the serum calcium level. In the opposite case, when a pathophysiological extra-renal source of CYP27B1 (e.g., the macrophage in granuloma-forming diseases; see right panel, Figure 1 and Table 1) becomes dysregulated and dominant, serum 1,25(OH)_2_D-driven hypercalcemia and/or hypercalciuria [1,25(OH)_2_D intoxication] occurs overriding the calcium-lowering actions of the CaSR in the parathyroid gland and in the kidney (57). Only in these two abnormal calcemic states is the serum level of 1,25(OH)_2_D of the host informative to the clinician in evaluating a patient.

At the molecular level, 1,25(OH)_2_D binds VDR with the highest affinity among all vitamin D metabolites regardless of whether 1,25(OH)_2_D is coming from the serum outside the target cell (endocrine mode), from the local microenvironment outside of the serum compartment (paracrine mode), or from the inside of the cell (intracrine mode) (58). In the 1,25(OH)_2_D-liganded state the VDR preferentially forms heterodimers with retinoic acid X receptor (RXR). In the 1,25(OH)_2_D-occupied state the VDR and its unliganded RXR partner heterodimer become transacting complexes binding to specific cis-acting vitamin D response elements (VDREs) in the genome. The heterodimer interacts with the transcriptional machinery resulting in 1,25(OH)_2_D-regulated (positive or negative) gene expression and corresponding bioactivities. Due to the three-dimensional “looping” nature of DNA-protein interactions, VDRE-like enhancer and repressor motifs can found at considerable distance from the transcriptional start site of a vitamin D regulated gene (59, 60).

Owing to a rapidly growing skeleton with a high demand for calcium and phosphate for skeletal mineralization and in the face of normal VDR and CYP27B1 activity, children suffering from low 25(OH)D levels and secondary decreases in optimal intestinal absorption in dietary calcium and phosphate can develop vitamin D deficient rickets (61). This is commonly observed in subpopulations of impoverished, dark-skinned children (i) who require up to 10 times more cutaneous sunlight exposure that lightly pigmented children to make the same amount of vitamin D in their skin and (ii) in whom consumption of natural vitamin D-rich foods (e.g., fish) and vitamin D supplemented foods is compromised. In nations of lower income, children usually consume a diet rich in grains; grains are a rich source of phytates known to chelate ingested calcium further decreasing intestinal calcium absorption. Vitamin D deficiency rickets can also be hastened in children subjected to religious/cultural practices that (e.g., occlusive garb) that effectively eliminate skin exposure to sunlight. In vitamin D deficient rickets the serum calcium and phosphate is low and PTH and 1,25(OH)_2_D usually elevated for the subject's age (62). A low 25(OH)D level (usually <10 ng/ml) is the distinguishing marker for vitamin D deficient rickets, distinguishing it from Human Vitamin D Resistant Rickets (HVDRR; high 1,25(OH)_2_D) and Pseudo Vitamin D Deficient Rickets (PDDR; low 1,25(OH)_2_D) (63). Appropriate dietary supplementation with calcium and vitamin D to normalize the serum 25(OH)D level (e.g., >30 ng/ml) alleviates nutritional rickets (61, 64).

Prevention of nutritional rickets in children and osteomalacia in adults is the primary concern behind the recommendations on vitamin D intake and serum 25(OH)D level attainment. Various medical organizations have evaluated the existing body of research data to support their positions and are exhaustively reported elsewhere (45, 48). However, two key studies are emblematic of the basis for the determinations. In one line of evidence, PTH is used as a biomarker of bone health. In some studies, an inverse association between PTH (declining) and 25(OH)D (increasing) has been observed (55, 65, 66). In one study involving 1,536 post-menopausal women (55) an inflection point where the decline in serum PTH levels off was identified at a 25(OH)D level of 30 ng/ml, leading some authorities in the vitamin D field to call for this to demarcate the cutoff for vitamin D sufficiency (46). Another often quoted study involved post-mortem (*N* = 675) determination of 25(OH)D levels and comparisons to histomorphometric analysis of transiliac crest biopsies (67). From this dataset, the IOM (45) concluded that at a serum 25(OH)D level of 20 ng/ml and above, 99% of the normal population (e.g., without a known bone disease) should have no pathological accumulation of osteoid (unmineralized portion of bone indicative of rickets in children and osteomalacia in adults). Interestingly, the original authors (67) using different criteria to analyze the same data concluded that 30 ng/ml 25(OH)D as the recommended level for optimal skeletal health.

## Total 25(OH)D or Free 25(OH)D?

The measured total 25(OH)D concentration in serum is present at nearly 1,000-fold higher levels compared to 1,25(OH)_2_D in serum and easily and accurately measured from a small amount of sample (25–50 μl) (68). As such, total 25(OH)D has become the de facto biomarker of the state of vitamin D deficiency or sufficiency of the host. However, the majority (>99%) of serum 25(OH)D is bound (Figure 1, left box) to carrier proteins (~85% to vitamin D binding protein [DBP]; ~15% to albumin). The affinity of DBP for 25(OH)D is ~1,000-fold greater than that of albumin for 25(OH)D (11). Cells that express the cell surface receptor proteins megalin and cubulin can internalize the DBP-bound-25(OH)D complex into an endolysosome with ultimate release of 25(OH)D from DBP into the cell interior for further metabolism and/or catabolism of 25(OH)D. This mechanism of endocytosis, intracellular release of 25(OH)D from acid-hydrolyzed DBP has been most clearly demonstrated at the luminal membrane of the tubular epithelial cells in the kidney (15). Here internalized 25(OH)D is the substrate used by the CYP27B1 to form 1,25(OH)_2_D for endocrine distribution. In cells not expressing megalin, 25(OH)D entry into its target cell is proposed to be accomplished by diffusion of the unbound, free 25(OH)D across the lipid bilayer of the plasma membrane to the cell interior (15). Thus, the biologically relevant substrate for some cells is DBP-bound 25(OH)D (essentially equal to total 25(OH)D) and for others it is the free 25(OH)D or a related metric of bioavailable 25(OH)D (sum of free 25(OH)D and albumin-bound-25(OH)D).

Though the serum protein carriers of vitamin D metabolites are well-characterized, it is now clear that vitamin D metabolites also reside in non-serum locales such as body fat and inside cells, suggesting that specific vitamin D binding proteins other than DBP and albumin may also play a role in vitamin D biology. Notably intracellular vitamin D binding proteins (IDBPs) were identified as a result of investigations into apparent vitamin D resistance in New World Primates [NWP; reviewed in Adams et al. (69, 70)]. The high serum levels of steroid hormones in general and vitamin D in particular, relative to Old World Primates (OWP), was shown to be associated with a form of target tissue insensitivity to 1,25(OH)_2_D (71). The first indications of a functional role for IDBPs was the observed diminished ability of NWP cells to effectively upregulate VDR-target genes, like the *CYP24A1*, despite having comparable amounts of VDR (72, 73) and VDR functionality (74). Using cell extracts from NWP and OWP in VDR-1,25(OH)_2_D radiolabel binding assays, a protein in NWP cell extracts was observed to prevent VDR-1,25(OH)_2_D binding when mixed with OWP extracts. This inhibition of binding was abolished after trypsin digestion or heat denaturation (75). Upon further characterization, IDBP was found to (i) bind 25(OH)D as well as other steroid hormones (76) and (ii) be part of the heat shock protein 70 family (77, 78). With cDNA constructs for IDBP obtained, transient and stable overexpression *in vitro* in tissue culture studies revealed that IDBP could increase 1,25(OH)_2_D synthesis, possibly by chaperoned delivery of 25(OH)D to the CYP27B1 (79). The ATPase domain of IDBP was essential to this function (80, 81), with the BCL2-associated athanogene 1 (BAG1) serving as an IDBP co-chaperone (82).

Measurement of the free, unbound form of 25(OH)D in serum is challenging, because its levels are low (4–8 pg/ml range) and has historically required radioactive tracers of 25(OH)D with equilibrium or centrifugal dialysis methods that are cumbersome and impractical for use in clinical laboratory testing services (10, 11). Free and bioavailable 25(OH)D levels in the serum can be mathematically calculated using equations that incorporate the binding affinity of DBP for 25(OH)D and the concentration of DBP in the serum (11, 83). This approach of calculating the free, biologically active fraction of 25(OH)D has also been taken with testosterone (84) and thyroid hormone (85). Unfortunately, for free 25(OH)D, it was discovered that one commonly used DBP ELISA kit used to calculate for free 25(OH)D had differential sensitivities to the common phenotypic variants of DBP (discussed later in this review) that resulted in inaccurate measurement of DBP concentrations in some samples leading to inaccurate calculated free 25(OH)D levels in those serum samples.

Direct measurement of the various free hormones of clinical importance have been developed (86, 87). However, this aspect of clinical chemistry remains challenging due to (i) the low concentration of these metabolites in the serum (Table 3) and (ii) variability of serum composition (e.g., lipids and other potentially interfering molecules) between patients that can introduce error into the measured value. Recently, an ELISA based method for detection of free 25(OH)D has been developed (90) and has been utilized in a number of clinical studies that we will summarize in the clinical studies portion of this review. Most recently, a high-throughput method to measure bioavailable 25(OH)D has been developed (91). However, procedures to ensure accuracy and precision across a wide range of sample conditions both for both of these assays have not been yet developed; as a consequence, these assays are deemed for research use only.

**Table 3:** Hormone binding proteins found in human serum.

**Binding protein**	**Metabolites**		**Percent free**
DBP 4.5–5.5 μM^a^	Total 25(OH)D 25–75 nM^a^	Free 25(OH)D 5–20 pM^a^	0.02
	Total 1,25(OH)_2_D 50–198 pM^b^	Free 1,25(OH)_2_D 325–525 fM^c^	0.5
TBG 241–722 nM^b^	Total T4 58–154 nM^b^	Free T4 11–23 pM^b^	0.02
	Total T3 1.1–2.8 nM^b^	Free T3 3.0–6.8 pM^b^	0.3
SHBG 16.5–55.9 nM^b^(male)	Male total T 9.2–31.8 nM^b^	Male free T 30–87 pM^b^	0.3
	Male total E2 28–156 pM^b^	Male free E2 <1.7 pM^d^	1
SHBG 24.6–122.0 nM^b^ (female)	Female total T 0.3–1.7 nM^b^	Female free T <15 pM^b^	0.9
	Female total E2 46–609 pM^b^	Female free E2 1.6–18.5 pM^d^	3

a *Nielson et al. (88)*.

b *https://www.labcorp.com/test-menu/search*.

c *Bikle et al. (89)*.

d *https://www.questdiagnostics.com/testcenter/TestCenterHome.action*.

*Vitamin D binding protein (DBP), thyroxine binding globulin (TBG), and sex hormone binding globulin (SHBG) are tabulated with their serum concentration levels. The respective metabolite concentrations total, free and percent free are also presented. Albumin can bind all the hormones listed and transthyretin can bind T4 albeit at lower affinity compared to their primary carrier proteins. The bioavailable concept has been applied to these hormones and comprise the sum of albumin-bound-hormone and free hormone*.

## Molecular Biology and Biochemistry of DBP

The vitamin D binding protein (DBP) is a multi-functional protein also known as Group Specific Component (GC) [reviewed in Chun (92) and Delanghe et al. (93)]. DBP is a member of the albumin superfamily of proteins. DBP is a moderately abundant protein (~5 μM in humans) in serum of vertebrates. In humans the *GC/DBP* 13 exon *gene* is located at 4q13.3. Its gene product, DBP, is highly expressed in the liver and exported into the circulatory system. DBP contains 474 amino acids of which the 16 N-terminal amino acids function as a signal peptide. DBP can be glycosylated (94) to varying degrees depending on genotype. The glycosylation pattern has been suggested to be structural basis for the macrophage activating factor activity of DBP (95, 96).

DBP's most well-characterized role is that of a carrier protein for vitamin D metabolites. Its rank order avidity for the vitamin D and it metabolites are (10, 11, 89, 97) as follows: 24,25(OH)_2_D>25(OH)D>1,25(OH)_2_D>vitamin D. DBP binds vitamin D3 and its metabolites with greater affinity than vitamin D2 and it metabolites (97). DBP can also bind actin (98). This biological action is suggested to be that of scavenging of exposed actin, preventing overzealous extracellular polymerization after tissue injury (99). DBP can also bind circulating fatty acids (100) and C5a des Arg, the latter of which enhances complement activation (101).

DBP migrates at ~52–59 kDa in electrophoretic gels (102). It was variations in DBP mobility in isoelectric focusing (IEF) gels that garnered initial research attention prior to its function being determined. One banding pattern was termed GC-1F (faster), another GC-1S (slower), and still another GC2 (103). GC2 migrated less rapidly toward the anode compared to either GC1 forms. The different forms, due to single amino acid differences (Table 4), were (i) used to determine allelic frequency for samples worldwide (104) and (ii) found to associate with the racial background of the source human serum. In the Kamboh study, black subjects were more likely to have the GC1F forms (67–79% of alleles in USA blacks) while white subjects more frequently yielded the GC1S pattern (49–57% of alleles in USA whites). The GC2 allele was observed were more frequently seen in white subject samples (21–31% in USA whites) compared to blacks (8–13% in USA blacks). These three classic forms (GC1F, GC1S, and GC2) account for the vast majority of the variation DBP across human populations. Since these early studies, many other SNPs (105) have been found in GC of which a small percentage encodes a missense mutation that changes the amino acid code.

**Table 4:** Molecular biology of most common DBP polymorphisms.

**SNP name**	**GC name**	**Codon variant**	**Amino acid variant**
rs4588	GC1	ACG	Thr-436
	GC2	AAG	Lys-436
rs7041	GC1F	GAT	Asp-432
	GC1S	GAG	Glu-432

*Two single nucleotide polymorphisms (SNP) account for three of the major forms of DBP (originally known as GC-globulin). Table also includes the specific codon and amino acid variation that define these variants*.

### Affinity Differences and DBP Genotype

The biological significance of DBP's different allelic forms is unclear. Conceptually, if the different genotypic forms of DBP had different affinities for vitamin D metabolites, then the levels of those metabolites could be influenced by the genotype. However, in terms of experimentally measured differences in affinity for vitamin D metabolites, one study reported large differences (106) but three others did not (107–109). Media supplemented with serum that contained different alleles of DBP had differing impacts on assessed immunological readouts consistent with affinity differences in three *in vitro* studies (110–112). The mechanism for the observed differences were not investigated in those studies; thus, non-affinity dependent mechanisms cannot be ruled out. Using an ELISA based assay to detect free 25(OH)D, some small differences in percent free were detected between the genotypes (113). However, these differences were smaller in magnitude than the differences anticipated if the report of large affinity difference among genotype was accurate.

### DBP Serum Concentration and DBP Genotype

The other mechanism by which DBP genotype could impact free 25(OH)D levels is through differentials in the genotype-dependent DBP concentration. It is on this basis that Powe et al. (114) proposed how black Americans, with lower total serum 25(OH)D levels and substantially lower DBP levels compared to white Americans, could ultimately have similar free 25(OH)D levels. However, it was eventually determined that the monoclonal antibody-based ELISA used in their study was less sensitive to the GC1F DBP, the form most frequently found in blacks (88, 115). This resulted in an underestimation of serum DBP levels in black subjects and a consequent overestimation of the calculated free and bioavailable serum 25(OH)D levels in their serum. Nonetheless, there is evidence of some genotypic effect on DBP concentrations. One early study (116) of Danish women found DBP concentration ranked according to the presence of GC1 alleles (GC1-GC1 > GC1-GC2 > GC2-GC2). Consistent with those findings was a study in a population male subjects with modest racial diversity where GC2/GC2 subjects exhibited the lowest serum DBP concentration (88).

In another recent study utilizing serum samples from both women and men with modest racial diversity, the presence of the GC2 allele in one of three allelic combinations resulted in lower DBP levels compared to the three allelic combinations without GC2 (113). Currently, it is thought that DBP's role in determining free 25(OH)D levels is largely through its concentration [i.e., for a fixed total 25(OH)D, more DBP yields less free 25(OH)D] and perhaps to a small degree by genotype. As such, in most cases total 25(OH)D and free 25(OH)D are highly correlated. However, there are some conditions where DBP levels diverge from typical levels. For instance, patients with liver disease have lower levels of DBP while pregnant women have higher DBP levels (89, 117) resulting in higher free 25(OH)D levels in those with lower DBP concentrations. In the more extreme case of patients before [lower DBP, lower 25(OH)D] and after [higher DBP, higher 25(OH)D] liver transplants, the percentage of free 25(OH)D relative to total 25(OH)D is higher before transplantation. Recently, a case report described a subject with a homozygous deletion of DBP (118). Compared to unaffected and heterozygous siblings, the affected patient had no DBP and very low serum levels of 25(OH)D and 1,25(OH)_2_D. Despite the extremely low serum levels of vitamin D metabolites, the subject had normal calcium balance with only relatively small alterations in bone health and mineral metabolism. This case report is the strongest evidence to date that the total serum 25(OH)D and 1,25(OH)_2_D level can be disconnected from “normal” vitamin D status. One explanation is that albumin, or some other chaperone in the serum, assumes the role of delivering 25(OH)D and maintaining viable levels of free 25(OH)D and 1,25(OH)_2_D, as was measured in this subject, to target tissues for further metabolism and action.

## Animal Studies of Free 25(OH)D and Bone

*Dbp* heterozygous and homozygous knockouts in mice do not have any obvious bone phenotype relative to wild type (119). However, with the loss of a single copy of *Dbp* the levels of circulating total serum 25(OH)D and 1,25(OH)_2_D are diminished and when both copies were absent, the levels of serum 25(OH)D and 1,25(OH)_2_D were extremely low, phenocopying the *DBP*^−/^^−^ subject just described above. Interestingly, neither DBP^+/−^ or DBP^−/−^ mice exhibited any skeletal abnormalities or problems with calcium and phosphate balance (119). These findings support the hypothesis that free hormone levels alone are adequate for sustaining skeletal health. Consistent with this was another study with *Dbp* homozygous knockouts where the levels of 1,25(OH)_2_D was measured in the intestinal tissues (120). Even though the double knockout animals had very low total serum 1,25(OH)_2_D levels compared to wild type, the levels of 1,25(OH)_2_D measured in the intestinal tissues were very similar. These findings suggest that trace amounts of free metabolites and/or enhanced local conversion of 25(OH)D to 1,25(OH)_2_D are sufficient as long as sufficient amounts substrate 25(OH)D are available to the host. The fact that mice with no DBP are still viable suggests that albumin, though having a much lower affinity for vitamin D metabolites, could serve as the carrier protein in place of DBP. When *Dbp* null mice were raised on vitamin D3-free diets, they developed secondary hyperparathyroidism and bone mineralization defects much more rapidly than paired wild-type mice (119). This result indicated that DBP's function is to maintain a stable reservoir of circulating extracellular vitamin D metabolites. Owing to its higher affinity for vitamin D metabolites, DBP is more effective in this role than albumin, despite both DBP and albumin being filtered into the urine and reclaimed by megalin.

Another test of the biological impact of free 25(OH)D in mice (121) was based on the difference in affinity of DBP for D2 or ergocalciferol (vitamin D found in fungi) vs. D3 or cholecalciferol (vitamin D found in animals) forms of vitamin D metabolites. Mice raised on diets containing exclusively D2 or D3 would result in the mice having only 25(OH)D2 and 25(OH)D3 circulating in their serum. Since DBP affinity for D2 forms is lower relative to D3 forms, the free 25(OH)D2 levels were expected to be higher in animals raised on D2 diets. In this study, mice were placed on D2 or D3 diets (1,000 IU/kg) beginning at week 3 and tested at week 8 and week 16 for 25(OH)D3 and 25(OH)D2 serum levels and bone phenotype by histomorphometry. These mice had similar total 25(OH)D levels at week 8 (26.6 ± 1.9 ng/ml 25(OH)D2 vs. 28.3 ± 2.0 ng/ml 25(OH)D3) and at week 16 (33.3 ± 4.4 vs. 31.7 ± 2.1 ng/ml). However, as anticipated, they differed in their free 25(OH)D levels with free 25(OH)D2 greater than free 25(OH)D3 at week 8 (16.8 ± 0.65 vs. 8.4 ± 0.63 pg/ml, *P* < 0.001) and at week 16 (17.4 ± 0.43 vs. 8.4 ± 0.44, *P* < 0.001). Histomorphometric analysis of their bones detected that at week 8, the D2 fed mice had significantly higher osteoclast surface/bone surface, eroded surface/bone surface, and mineral apposition rate (high bone turnover) compared with mice raised on the D3 diets. Additionally, osteoblast surface/bone surface, an index of bone formation, was higher in week 8 D2 in females only. The reason underpinning this sexual dimorphism in bone formation rates remains unknown. The bone phenotype at week 16 revealed significantly higher bone volume/total volume and trabecular number in the D2 mice relative to the D3 mice. Taken together, despite similar total serum 25(OH)D levels, bone phenotype differences were observed in association with different free 25(OH)D levels (higher in D2 mice) suggesting the relevance of free 25(OH)D to bone health.

To the best of our knowledge, there have been no studies on the effects of free 25(OH)D and the bone health of animals besides the studies in mice summarized above. However, the existence of nocturnal bats that roost in dark locations (122) could be informative. These bats have very low total serum 25(OH)D (<5 ng/ml) as their normal state suggesting that “deficient” (by human standards) total 25(OH)D levels presumably still yields adequate free 25(OH)D levels to sustain normal bone and mineral homeostasis for this species. Interestingly, these bats are fully capable of attaining higher 25(OH)D levels when housed in conditions that expose them to sunlight. Perhaps these animals have lower DBP concentrations or metabolite affinities for DBP to compensate for their naturally low total serum 25(OH)D levels. A test of these animal's serum with the free 25(OH)D assay could be informative. Other possible adaptations that permit compensation to the very low circulating levels of 25(OH)D include: (1) a highly efficient CYP27B1; (2) hyper-sensitive VDR to ligand; (3) heightened transactivation potential of VDR-interacting co-activators; and/or (4) diminished functional activity of the CYP24A1 catabolic machinery. There is one study comparing DBP affinity for 25-hydroxyvitamin D among several animals; these investigators found the DBP from rat and cattle exhibited higher affinity to 25(OH)D compared to horse and rhesus monkey with humans having the lowest affinity of species tested (123). What the levels of free 25(OH)D are and its importance to these species have not yet been examined.

## Human Studies of Free 25(OH)D

Revitalized interest on the impact of vitamin D on human health beyond bone was spurred by studies that investigated vitamin D's regulatory role in the adaptive and innate immune system (26, 29, 124). Three *in vitro* studies have demonstrated that decreasing free 25(OH)D by increasing DBP in the culture media diminished immune functions of adherent monocytes (110), dendritic cells (111), and T-lymphocytes (112). Because of these findings *in vitro*, the parameter of free 25(OH)D began to receive greater interest in clinical research. In non-bone health association studies, the results have been mixed. In some studies free serum 25(OH)D levels were inversely associated with coronary artery disease (125), pediatric inflammatory bowel disease (126) and ulcerative colitis (127), insulin sensitivity (128), reduction in lipid markers in statin patients (129), and acromegaly (130). However, in some other studies free 25(OH)D was inferior in asthma (131) and no better than total 25(OH)D for colorectal cancer in African-Americans (132).

Concerning bone and mineral health, a 2011 report (133) was the first to utilize free 25(OH)D and the related concept bioavailable 25(OH)D (sum of free 25(OH)D and albumin-bound 25(OH)D) for analytical purposes. They found that these two metrics of serum vitamin D status were more closely and directly associated with BMD in the individual than total serum 25(OH)D. This group then followed up with another report (134) that showed that compared to measures of total 25(OH)D bioavailable 25(OH)D had a significant direct association with serum calcium (corrected for the serum albumin level) and a significant inverse association with PTH. These investigators suggested that this could address the long-standing paradox of how black Americans with lower total serum 25(OH)D levels have higher BMD and similar PTH levels compared to white Americans. In additional work, they measured DBP serum concentrations in black Americans and found them to be lower than those in white Americans. They concluded that the resultant higher bioavailable 25(OH)D may account for the nominal differences in BMD and serum PTH (114) despite what appeared to be sub-optimal total serum 25(OH)D levels; unfortunately, a high-throughput assay to directly measure free 25(OH)D was not available at that time. Thus, in these reports, measured DBP, albumin and total 25(OH)D values were input into mathematical equations (11, 84) to calculate values for free 25(OH)D and bioavailable 25(OH)D. Disappointingly, one of the popular ELISA kits for DBP quantitation at the time relied upon a monoclonal antibody that turned out to possess reduced sensitivity to the DBP polymorphism (GC1F) most frequently found in black Americans (88, 115). Because of this characteristic of the ELISA, the DBP concentration was under-reported for these subjects leading to an over-estimation of the calculated free and bioavailable 25(OH)D. The manufacturer of this monoclonal ELISA has re-designed their ELISA and released a new version in January 2017 addressing this problem (135). Even with these complications, these early studies sparked interest in examining bone and mineral health by markers of vitamin D status other than the traditional total serum 25(OH)D.

Investigators have continued to use calculated free and bioavailable 25(OH)D in serum in association studies. In light of the difficulties with the monoclonal antibody-based ELISA for DBP, immunological methods based on polyclonal antibodies (less influenced by DBP polymorphisms) and techniques independent of antibodies entirely (mass spectrometry) can be employed to measure DBP for calculation of bioavailable 25(OH)D in serum (88, 115). Additionally, an ELISA method has been developed that measures free 25(OH)D directly (90) such that some investigators use this assay exclusively in their studies though many also include data from calculated free and/or bioavailable 25(OH)D.

## Human Clinical Studies of Free 25(OH)D and Bone

This section surveys the recent literature examining whether free (directly measured or calculated using the polyclonal DBP assay) vs. total 25(OH)D is more consistently associated with various measures of bone health, including intestinal calcium absorption, parathyroid hormone secretion, and bone mineral density.

### Intestinal Calcium Absorption

To our knowledge, there has only been one study to date examining the relation between free and total 25(OH)D with intestinal calcium absorption. Aloia et al. randomized 71 adults to receive either placebo, 800, 2,000, or 4,000 IU/days of vitamin D3 over 8 weeks. At both baseline and follow-up, neither free nor total 25(OH)D nor 1,25(OH)_2_D in the serum was associated with intestinal calcium absorption efficiency (136). This supports the concept that VDR-directed increases in intestinal calcium absorption are controlled locally, outside of the serum compartment.

### Parathyroid Hormone

Multiple investigators have assessed whether free vs. total 25(OH)D is more strongly correlated with the serum PTH level, with results being inconsistent. For example, in an analysis of 155 subjects that included 24 cirrhotics and 20 pregnant women, Schwartz et al. reported that both free and total serum 25(OH)D were similarly, inversely correlated with the serum PTH (117). Similar findings have been reported in: (1) healthy pre- (137) and postmenopausal women (136); (2) individuals with obesity (138); (3) patients cirrhosis of the liver (139); (4) children (2–18 y/o) in Spain (140); (5) blacks and whites in a supplementation study (placebo, 2,000, 4,000 IU/days for 16 weeks) (141); (6) a RCT of prediabetics (142); and (7) pregnant white women in Germany (143). While the above studies reported similar correlations between serum free and total 25(OH)D with serum PTH, others have favored free 25(OH)D. For example, Schwartz et al. conducted a 16-weeks trial in which 81 older women and men received vitamin D3 at doses of 800, 2,000, or 5,000 IU/days or 50,000 IU/weeks. At the end of the study, free, but not total serum 25(OH)D was inversely associated with the serum PTH; however, free 25(OH)D explained only a small amount of the variability in PTH [*R*^2^ = 0.08; (144)]. In two, smaller trials – one in which 38 participants received 500,000 IU of vitamin D2 or D3 over 10 weeks (145) and another in which 35 participants received 2,400 IU/day of vitamin D3 or 20 mcg/day of 25-hydroxyvitamin D3 (146)—Shieh et al. reported that longitudinal increase in free 25(OH)D was significantly associated with concurrent decrease in serum PTH during the first 8–10 weeks of supplementation (when 25(OH)D levels change most rapidly), whereas increase in total 25(OH)D was not. In adults with primary hyperparathyroidism, Wang et al., similarly found that the free serum 25(OH)D was inversely correlated with circulating PTH levels, but total 25(OH)D was not (147). Further complicating the picture are studies favoring total 25(OH)D over free 25(OH)D. In a cohort of Hungarian adults assessed at the end of winter total, but not free 25(OH)D, was inversely correlated with PTH (148). In a study of UK whites and south Asians, total, but not free 25(OH)D, was inversely correlated with PTH (149). In a study of pregnant adolescents (13–18 y/o), the inverse association of PTH with free 25(OH)D was weaker than that observed total 25(OH)D (150).

### Bone Mineral Density

While a change in the serum PTH level is the outcome that has been most frequently tested in relation to free vs. total 25(OH)D, some cross-sectional studies have examined bone mineral density (BMD) as well. As was the case with changes in the serum PTH, results with BMD have been inconsistent. For example, Jemielita et al. reported that in 304 adults, neither total nor free serum 25(OH)D at a single point in time was associated with BMD assessed by DXA, or peripheral quantitative CT (151). In contrast, in a cross-sectional analysis comparing the correlations between free vs. total serum 25(OH)D with BMD and composite indices of femoral neck strength, Alwan reported that higher free 25(OH)D levels were correlated with greater BMD (lumbar spine, femoral neck, total hip), and femoral neck strength (*r* = 0.24–0.34, *p* < 0.05), but total 25(OH)D was not (152). On the flip side, Michaelsson et al. reported that in women from Sweden (mean age 68 years) higher total, but not free, serum 25(OH)D was associated with greater BMD (153).

## Challenges and Prospects

Undoubtedly, further clinical studies should be conducted using bone/mineral outcomes as well as non-skeletal health readouts to assess the utility of free 25(OH)D. As described in the above section pertaining to bone and mineral health, human studies comparing whether free vs. total serum25(OH)D is more frequently associated with intestinal calcium absorption, parathyroid hormone secretion, or bone mineral density have yielded inconsistent results ranging from no difference, to those favoring either free, or total 25(OH)D. We propose that there are two major challenges that contribute to these inconsistencies. First, is the lack of a specific “readout” of vitamin D bioactivity. While circulating PTH levels are influenced by vitamin D status (154–156), it is also regulated by the calcium sensing receptor. Additionally, attempts to associate BMD with vitamin D status are complicated by the fact that BMD in adults is principally determined by attained peak bone mass that is partly dependent on vitamin D status (157). Thus, it is difficult to discern the relative importance of free vs. 25(OH)D in human cross-sectional studies using these parameters. As such, even when studies report statistically significant correlations, the r values tend to fall in the range (−0.3–0.1 and 0.1–0.3) that are deemed “weak relationship” by statisticians. Second, many human studies do not employ subjects who are 25(OH)D deficient. These subjects would have the most to gain physiologically from treatment to return 25(OH)D in the serum to normal. Ideally, supplementation studies must include subjects that are clearly at insufficient levels and then have it demonstrably shown that their levels are raised into the sufficient range. It is through longer-term longitudinal analyses of intra-individual changes in serum total and free 25(OH)D after an aggressive vitamin D supplementation regimen that would have a greater likelihood to detect any associations.

Lastly, there is currently only one method of direct measurement of free 25(OH)D (90) with reasonable throughput. Though very promising, this assay is based on antibody interaction with 25(OH)D and needs further validation on the wide variety of sample quality (i.e., time from collection to testing, temperature of storage, variability of potentially interfering serum components among patients, etc.) encountered in clinical laboratory practice (158). In the future, perhaps a mass spectrometry-based method could be developed as is occurring in the measurement of other free hormones such as estradiol (159), thyroid hormone (160), and testosterone (161).

## Author Contributions

JA developed the overall organization of and approved this publication. JA, RC, AS, CG, and MH wrote sections of this review. VY and JW participated in copy editing.

### Conflict of Interest

The authors declare that the research was conducted in the absence of any commercial or financial relationships that could be construed as a potential conflict of interest.
